# Congruent microbiome signatures in fibrosis-prone autoimmune diseases: IgG4-related disease and systemic sclerosis

**DOI:** 10.1186/s13073-021-00853-7

**Published:** 2021-02-28

**Authors:** Damian R. Plichta, Juhi Somani, Matthieu Pichaud, Zachary S. Wallace, Ana D. Fernandes, Cory A. Perugino, Harri Lähdesmäki, John H. Stone, Hera Vlamakis, Daniel C. Chung, Dinesh Khanna, Shiv Pillai, Ramnik J. Xavier

**Affiliations:** 1 Broad Institute of MIT and Harvard, Cambridge, MA USA; 2 Department of Computer Science, Aalto University, 02150 Espoo, Finland; 3 Novartis Institute for Biomedical Research, Cambridge, MA USA; 4 Division of Rheumatology, Allergy, and Immunology, Massachusetts General Hospital, Boston, MA USA; 5 Clinical Epidemiology Program and Rheumatology Unit, Massachusetts General Hospital and Harvard Medical School, Boston, MA USA; 6 Ragon Institute of MGH, MIT and Harvard, Cambridge, MA USA; 7 Division of Gastroenterology, Massachusetts General Hospital and Harvard Medical School, Boston, MA USA; 8 Center for Cancer Risk Assessment, Massachusetts General Hospital and Harvard Medical School, Boston, MA USA; 9 University of Michigan Scleroderma Program, Ann Arbor, MI USA; 10 Center for Computational and Integrative Biology, Massachusetts General Hospital and Harvard Medical School, Boston, MA USA; 11 Department of Molecular Biology, Massachusetts General Hospital and Harvard Medical School, Boston, MA USA; 12 Center for Microbiome Informatics and Therapeutics, MIT, Cambridge, MA USA

**Keywords:** Gut microbiome, IgG4-RD, Systemic sclerosis, Autoimmunity

## Abstract

**Background:**

Immunoglobulin G4-related disease (IgG4-RD) and systemic sclerosis (SSc) are rare autoimmune diseases characterized by the presence of CD4+ cytotoxic T cells in the blood as well as inflammation and fibrosis in various organs, but they have no established etiologies. Similar to other autoimmune diseases, the gut microbiome might encode disease-triggering or disease-sustaining factors.

**Methods:**

The gut microbiomes from IgG4-RD and SSc patients as well as healthy individuals with no recent antibiotic treatment were studied by metagenomic sequencing of stool DNA. De novo assembly-based taxonomic and functional characterization, followed by association and accessory gene set enrichment analysis, were applied to describe microbiome changes associated with both diseases.

**Results:**

Microbiomes of IgG4-RD and SSc patients distinctly separated from those of healthy controls: numerous opportunistic pathogenic *Clostridium* and typically oral *Streptococcus* species were significantly overabundant, while *Alistipes*, *Bacteroides*, and butyrate-producing species were depleted in the two diseases compared to healthy controls. Accessory gene content analysis in these species revealed an enrichment of Th17-activating *Eggerthella lenta* strains in IgG4-RD and SSc and a preferential colonization of a homocysteine-producing strain of *Clostridium bolteae* in SSc. Overabundance of the classical mevalonate pathway, hydroxyproline dehydratase, and fibronectin-binding protein in disease microbiomes reflects potential functional differences in host immune recognition and extracellular matrix utilization associated with fibrosis. Strikingly, the majority of species that were differentially abundant in IgG4-RD and SSc compared to controls showed the same directionality in both diseases. Compared with multiple sclerosis and rheumatoid arthritis, the gut microbiomes of IgG4-RD and SSc showed similar signatures; in contrast, the most differentially abundant taxa were not the facultative anaerobes consistently identified in inflammatory bowel diseases, suggesting the microbial signatures of IgG4-RD and SSc do not result from mucosal inflammation and decreased anaerobism.

**Conclusions:**

These results provide an initial characterization of gut microbiome ecology in fibrosis-prone IgG4-RD and SSc and reveal microbial functions that offer insights into the pathophysiology of these rare diseases.

**Supplementary Information:**

The online version contains supplementary material available at 10.1186/s13073-021-00853-7.

## Background

The gut microbiome exists in an essential symbiosis with its human host by serving as a source of nutrients and small molecules, informing the development and activity of the immune system and providing colonization resistance against pathogens. Strains of gut bacteria stimulate the expansion of various immune cell populations [1] and provide signals for orchestrated anti- and pro-inflammatory responses locally and systemically [2]. Dysregulation of this symbiosis through expansion or depletion of specific taxa and their associated proteins and metabolic capabilities has been correlated with multiple diseases. In inflammatory bowel diseases (IBD) and rheumatoid arthritis (RA), microbiome studies yielded potential clues to the identity of specific microbes (e.g., *Ruminococcus gnavus*, *Prevotella copri*) that may act as sources of disease-triggering or disease-sustaining molecules and antigens.

Microbial antigens are recognized by the immune system via presentation on major histocompatibility complex (MHC) class II molecules to CD4+ T cells. Recently, an unusual subset of cytotoxic CD4+ T cells has been described in patients with two rare, fibrosis-prone autoimmune disorders: immunoglobulin G4-related disease (IgG4-RD) and systemic sclerosis (SSc) [3–5]. IgG4-RD and SSc are complex diseases characterized by chronic inflammation and generalized fibrosis in multiple organs as well as dysregulation of adaptive and innate immunity. IgG4-RD has been reported in almost every organ [6–8], with similar histopathological and serological features regardless of the disease site [6, 9, 10]. SSc, on the other hand, is a rare connective tissue disease that can be classified into four main subgroups: limited cutaneous SSc, diffuse cutaneous SSc, sine scleroderma, and overlap scleroderma [11]. These subgroups are determined based on the localization of the fibrosis, extent of skin involvement, circulating autoantibodies, and manifestation of other connective tissue diseases [11]. While both conditions can lead to failure of affected organs, IgG4-RD typically responds to therapy [10], while SSc has limited therapeutic options and is associated with high morbidity and mortality [12–14]. To date, the etiology and pathogenesis of each disease remain elusive and poorly understood.

The immunological characteristics of IgG4-RD and SSc overlap, where CD4+ T cells—including the unusual subpopulation of IFN-γ, IL-1β, and TGF-β secreting cytotoxic CD4+ T cells (CTLs)—play a key role in disease pathogenesis [4, 15]. Accordingly, they are the major constituents of the lymphoplasmacytic infiltrate in IgG4-RD and SSc lesions [3, 7, 16]. B cells have been implicated in pathogenesis by acting as antigen-presenting cells to CD4+ CTLs and producing different autoantibodies [17, 18]. Genetic studies in SSc have identified multiple single-nucleotide polymorphisms in the human leukocyte antigen (HLA) locus and in non-coding regions of the genome that strongly associate with the disease phenotype [15, 19]. In IgG4-RD, certain HLA haplotypes have been associated with autoimmune pancreatitis in Japanese and Korean populations [6, 7, 20]. Since the HLA genes encode MHC proteins that present antigens to CD4+ T cells, individuals with a certain genetic architecture could be predisposed to react to disease-triggering antigens. Antigens that are not exclusive to IgG4-RD and SSc have been implicated in the pathogenesis of each disease, including Annexin A11 [9] and galectin-3 [21]. These may stem from abnormal expression of intracellular proteins in damaged tissue after long-term exposure to toxic industrial chemicals [9, 11] or an encounter with specific microbes.

Given the pivotal role of the immune system in the pathogenesis of IgG4-RD and SSc, it is important to identify triggers of inflammatory responses and the potential contribution from the human gut microbiome. To our knowledge, there have been no reports on the microbiome in IgG4-RD, and only a few 16S studies in SSc described microbiome changes [22]. Here we evaluated stool microbiomes of patients with IgG4-RD and SSc, characterized their composition by metagenomics, and pinpointed strain differences and functional capabilities that distinguish them from a healthy control population. We detected consistent microbiome signatures in the two disorders that extend beyond known microbial species and include clades of unknown Firmicutes. These changes resemble microbiome signatures from other autoimmune diseases (e.g., *Eggerthella lenta* enrichment in RA) but not IBD. We also identified overabundance of microbiome pathways related to nutrition (ethanolamine utilization) and fibrosis (hydroxyproline utilization and fibronectin binding). Finally, strain-level analysis showed preferential colonization of autoimmune patients by *Clostridium bolteae* encoding a strain-specific cystine uptake and metabolism locus as well as by a proinflammatory strain of *E. lenta.*

## Methods

### Cohort and approval for human subject research

Patients with immunoglobulin G4-related disease (IgG4-RD, *N* = 58) and systemic sclerosis (SSc, *N* = 90) were recruited at Massachusetts General Hospital (MGH) and the University of Michigan, respectively. The IgG4-RD cohort included patients in remission (*N* = 45) and with active disease (*N* = 13) assessed using the IgG4-RD responder index [23]. SSc patients met the 2013 American College of Rheumatology/EULAR classification and were further subclassified as limited cutaneous SSc (lcSSc, *N* = 39), diffuse cutaneous SSc (dcSSc, N = 39), sine scleroderma (ssSSc, *N* = 7), and overlap scleroderma (osSSc, *N* = 5). Available patient information included age, sex, and current medication. As controls, healthy, non-medicated individuals (*N* = 165) were recruited during screening visits at MGH. For the purpose of this study, we only collected stool samples from the study participants. Human patient research in the IgG4-RD cohort was reviewed and approved by the Partners Human Research Committee (2008P002154). Human patient research in the SSc cohort, who meet the 2013 classification criteria, was approved by the Institutional Review Board of the University of Michigan Medical School (HUM00101836). Human patient research in the healthy control cohort was reviewed and approved by the Partners Human Research Committee (2015P000275). The study was approved by the Office of Research Subject Protection at the Broad Institute of MIT and Harvard. All experiments adhered to the regulations of these review boards. Study procedures were performed in compliance with all relevant ethical and federal regulations. This research conformed to the principles of the Helsinki Declaration. All study participants gave their written informed consent for sample collection and to participate in the study.

### Sample handling and sequencing

Stool samples were collected by study participants at home using self-collection kits in 100% ethanol and stored at room temperature for less than 48 h prior to dissection and long-term storage at − 80 °C, as previously described [24]. To extract nucleic acid from stool samples, we used the AllPrep 96 PowerFecal DNA/RNA kit from QIAGEN (custom product # 1114341). This method pairs bead-beating on a Tissuelyser II (QIAGEN) with a 96 well AllPrep protocol and is available through QIAGEN. Purified DNA was stored at − 20 °C. For metagenomic library construction, DNA samples were first quantified by Quant-iT PicoGreen dsDNA Assay (Life Technologies) and normalized to a concentration of 50 pg/μL. Illumina sequencing libraries were prepared from 100 to 250 pg of DNA using the Nextera XT DNA Library Preparation kit (Illumina) according to the manufacturer’s recommended protocol, with reaction volumes scaled accordingly. Prior to sequencing, libraries were pooled by collecting equal volumes (200 nl) of each library from batches of 96 samples. Insert sizes and concentrations for each pooled library were determined using an Agilent Bioanalyzer DNA 1000 kit (Agilent Technologies). Libraries were sequenced on HiSeq 2500 2 × 101 to yield ~ 10 million paired-end reads per sample. De-multiplexing and BAM and FASTQ file generation were performed using the Picard suite (https://broadinstitute.github.io/picard).

### Processing of sequencing data

The quality control for the metagenomic data was conducted using Trim Galore! to detect and remove sequencing adapters (minimum overlap of 5 bp) and kneadData v0.7.2 to remove human DNA contamination and trim low-quality sequences (HEADCROP:15, SLIDINGWINDOW:1:20), retaining reads that were at least 50 bp long. Metagenomic reads were assembled individually for each sample into contigs using MEGAHIT [25], followed by an open reading frame prediction with Prodigal [26] and retaining predicted genes that had both a start and a stop codon. A non-redundant gene catalog was constructed by clustering predicted genes based on sequence similarity at 95% identity and 90% coverage of the shorter sequence using CD-HIT [27, 28]. Reads were mapped to the gene catalog with the Burrows-Wheeler Aligner (BWA) requiring a unique, strong mapping with at least 95% sequence identity over the length of the read [29], counted (count matrix) and normalized to transcript-per-million (TPM matrix) using in-house scripts. Count matrix served as an input for binning genes into metagenomic species pan-genomes (core and accessory genes) using MSPminer with default settings [30]. We represented the abundance of every metagenomic species (MSP) in a sample as a median TPM for 30 top representative core genes reported by MSPminer. Assembled genes were annotated with KEGG KO genes [31] using eggNOG-Mapper [32] and at species, genus, and phylum levels with NCBI RefSeq (version May 2018) as described previously [33]. To annotate phylogenetically MSPs that had no match to any species from NCBI RefSeq, we used Phylophlan with default settings [34]. For reference-based microbiome analysis, we used MetaPhlAn v2.7.730 to determine relative abundance at species and phylum levels and HUMAnN2 v0.11.2 31 to functionally profile with MetaCyc pathways.

### Alpha and beta diversity calculations

Alpha diversities were calculated using Shannon and beta diversity was calculated using Bray-Curtis dissimilarity based on relative abundances at species and MSP levels (*R package vegan*). The significance of differences in alpha diversity between disease and control cohorts was determined using linear fixed effects modeling with covariates (age, sex, treatment information). Alpha diversity differences in IgG4-RD patients due to the disease activity and stratified by SSc subgroups were also evaluated using linear fixed effects modeling with age, sex, and treatment information as covariates.

### PERMANOVA analysis

The permutational multivariate analysis of variance (PERMANOVA) analysis was performed on the species-level data to identify correlation between the fixed effect covariates (age, sex, cohort information, and treatment information) and the composition of the gut microbiome as a whole. The PERMANOVA implementation in the *adonis()* function from the *vegan*-package in R was utilized in this study. A model selection step, determining the order of the linear predictors (fixed effect covariates) in the *adonis* function’s model formula, was performed prior to PERMANOVA analysis. Each covariate was individually analyzed for its association to the microbial dataset and then ordered based on the effect size (i.e., partial R-squared) from the most significant to the least. The pairwise distances between the species were determined using the Bray-Curtis dissimilarity measure and 1000 permutations were performed per analysis. Other parameters of the analysis were kept as default.

### Accessory genes analysis

We used USEARCH to identify, in the gene catalog, homologs of the 7 genes specific to *E. lenta*’s *cgr* locus [35], and detected strong hits for 5 of them at 95% sequence identity and 90% coverage (cgr1, cac1, cac3, cac4, cac5). An additional search for the missing hits to cgr2 and cac6 revealed that they assembled on the ends of the contigs as partial genes and hence were missing from the gene catalog (data not shown). We used a summed count of reads mapping to all 5 *cgr* locus gene homologs as a signal for abundance of the *cgr* locus. It is often difficult to disentangle the absence of a gene or operon in a bacterial genome from a missing observation due to an insufficient depth of sequencing. Yet the absence of a gene can readily be assessed when the counts of the core genes are proportional to the counts of the accessory genes, as identified by MSPminer [30]. Accordingly, the *cgr* genes can be reliably detected only in samples where the coverage of the *E. lenta* genome is sufficient. We used the proportionality between the *cgr* locus counts and the counts of the top 30 top representative core genes reported by MSPminer for *E. lenta* to derive the minimal number of reads mapping to *E. lenta* core genes that would allow the observation of at least 1 read mapping to the *cgr* locus. In that way, the metagenomic samples with less than 16 reads mapping to *E. lenta* core genes were discarded from the analysis. Odds ratios and significance of enrichment of the *cgr* locus in disease compared to controls were determined using a one-sided Fisher’s test. We note here that in the read mapping procedure, after the BWA step, we filter alignments to only retain uniquely mapped reads at 95% or greater level of nucleotide identity along the read length. This alleviates the risk of recruiting reads originating from identical or highly homologous regions in other genes.

For the microbiome-wide search for enriched accessory modules, we used a similar approach. A threshold of at least 1 mapped read was used to analyze the presence and absence of accessory modules reported by MSPminer and associated with specific MSPs; a minimal number of reads mapping to the top 30 representative core genes reported by MSPminer for a given MSP was similarly derived based on the proportionality rule to determine samples with enough signal for that MSP to be included in the analysis [30]. Additionally, we only considered MSPs detected in more than 20 healthy control, 20 IgG4-RD, and 20 SSc samples. Odds ratios and significance of enrichment of the accessory modules in disease compared to controls were determined using a two-sided Fisher’s test and nominal *P *values were adjusted for multiple hypothesis testing using Benjamini-Hochberg correction.

### Differential abundance analysis

Linear fixed effects modeling, as implemented in the *lm()* function from *stats-*package in R, was performed to identify differentially abundant features (metagenomic species, phyla, and various functional categories) between cohorts, SSc subgroups, and individuals with active and inactive IgG4-RD status. Prior to linear modeling, features present in less than 20% of the samples were filtered out. In analyses involving multiple cohorts, the less prevalent species (< 20% samples) that are specifically absent in either the control or disease cohorts (identified using Fisher’s exact test, FDR < 0.05) were included in downstream analyses. Furthermore, the zeros were replaced by half of the smallest non-zero measurement on a per-feature basis and log_10_ transformation was applied on the relative abundances for normality. In analyses studying differences between the cohorts (controls vs diseased, IgG4-RD vs SSc), linear modeling included fixed effect covariates: age, sex, cohort information and treatment information. Variable “treatment information” represented 6 different treatment categories: no treatment, rituximab (RTX), prednisone, other medication, RTX with prednisone and prednisone with other medication. Moreover, in analyses within a particular cohort, such as between individuals with the active and inactive IgG4-RD status and the SSc subgroups, covariates related to the IgG4-RD status and SSc subgroups were added to the respective models, while the cohort information was excluded. Comparison between the SSc subgroups and the control cohort was performed by expanding the cohort information with the four SSc subgroup classifications (i.e. classifying the samples belonging to the SSc category in the cohort-covariate into their respective subgroups). Nominal *P* values from the *lm()* output were adjusted for multiple testing using Benjamini-Hochberg correction and associations at FDR < 0.05 (unless stated differently) were considered as significant.

## Results

The autoimmune diseases IgG4-RD and SSc share a fibrotic phenotype and characteristic skew in immune cell populations. Both are associated with polymorphisms in the HLA locus and characterized by the presence of an unusual subset of cytotoxic CD4+ T cells [5], suggesting that dysfunctional immune recognition of microbial signals might underlie these pathologies. To identify potential sources of microbial signals, we sought to characterize the stool microbiome sampled at a single time point of patients diagnosed with IgG4-RD (*N* = 58) and SSc (*N* = 90), respectively recruited at Massachusetts General Hospital and University of Michigan (Table 1, Additional file 1: Table S1). The IgG4-RD cohort had similar numbers of males and females, while SSc patients were primarily female (82%). SSc patients were further classified across four common subgroups: limited cutaneous SSc (*N* = 39), diffuse cutaneous SSc (*N* = 39), sine scleroderma (*N* = 7), and overlap scleroderma (*N* = 5). Individuals in both disease cohorts had a similar age distribution (mean age ~ 60 years old) and approximately half were on treatment with immunosuppressive agents, such as prednisone and rituximab (RTX, mainly in IgG4-RD). A few individuals received a combination of drugs, such as RTX and prednisone or prednisone and another form of treatment (e.g., mycophenolate mofetil) (Table 1, Additional file 1: Table S1). Nearly a quarter of the IgG4-RD patients in this study had active disease at the time of sampling (*N* = 13, Table 1, Additional file 1: Table S1). As a control cohort, we used 165 healthy, non-medicated subjects (65% female) with age distribution similar to that of cases, recruited at Massachusetts General Hospital (Table 1, Additional file 1: Table S1).

**Table 1:** Characteristics of the IgG4-RD, SSc, and control cohorts used in the study. Other treatments include mycophenolate mofetil, methotrexate, hydroxychloroquine, abatacept, cyclophosphamide, azathioprine, tocilizumab, and etanercept. *RTX* rituximab, *lcSSc* limited cutaneous, *dcSSc* diffuse cutaneous, *ssSSc* sine scleroderma, *osSSc* overlap scleroderma

	Controls	IgG4-RD	Systemic sclerosis
*N* samples	165	58	90
*N* per subtype	na	Active: 13, inactive: 45	lcSSc: 39, dcSSc: 39, ssSSc: 7, osSSc: 5
Age, years (median ± sd)	57 ± 8	61 ± 13	57 ± 12
Males/females	58/107	29/29	16/74
RTX	0	16	1
Prednisone	0	7	27
Other treatment	0	3	44
Data collection center	Massachusetts General Hospital	Massachusetts General Hospital	University of Michigan

### Decreased diversity and a common microbiome architecture in IgG4-RD and SSc

To study the composition and function of the gut microbiome in IgG4-RD and SSc, we applied metagenomic sequencing to the stool samples collected, generating on average 24 M paired-end reads per sample. We taxonomically profiled the microbiomes using both reference-based (MetaPhlAn2 [36]) and de novo assembly-based (MSPminer [30]) methods, detecting 438 species and 504 metagenomic species (MSPs) respectively. The assembly-based microbiome profiling showed a higher alpha diversity compared to the reference-based profiling for all cohorts (*P* < 1 × 10^− 8^, Additional file 2: Figure S1), likely due to uncharacterized taxa that only an assembly-based approach can quantify. This motivated us to focus our taxonomic analysis on the assembly-based classification, while the reference-based profiling was only used for functional classification. Indeed, when considering taxonomic annotation of MSPs derived from a comprehensive and recent database of reference genomes (NCBI RefSeq), on average 26% of the relative abundance signal came from MSPs that had no species-level annotation (Additional file 2: Figure S2).

**Fig. 1:**
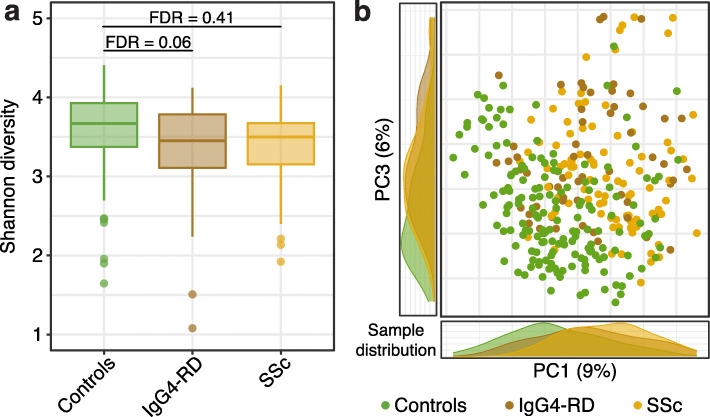
Microbiome community structure in IgG4-RD and SSc. **a** Alpha diversity boxplots indicate significantly lower alpha diversity in IgG4-RD compared to controls (FDR = 0.06). Boxplots show median and lower/upper quartiles; whiskers show inner fences. **b** PCoA plot of beta diversity using Bray-Curtis dissimilarity measure. Beta diversity correlates significantly with sample annotation according to IgG4-RD, SSc, or control status (PERMANOVA FDR = 0.004). Marginal figures present distributions of samples along each axis. Controls *N* = 165, IgG4-RD *N* = 58, SSc *N* = 90

Comparison of the Shannon diversity index revealed that IgG4-RD had a decreased alpha diversity compared to healthy controls (Fig. 1a, FDR = 0.06). While we did observe a similar trend in SSc patients, it was not statistically significant (Fig. 1a, FDR = 0.41). Rather, we observed a decrease in alpha diversity in SSc patients treated with oral prednisone in combination with other treatments (various disease-modifying antirheumatic drugs, DMARDs) (Additional file 1: Table S2, FDR = 0.001), indicating that anti-inflammatory medications can affect microbiome composition. No significant changes in alpha diversity were associated with different subgroups of SSc patients or IgG4-RD active disease status (Additional file 1: Table S2). To evaluate microbiome community structure differences between SSc or IgG4-RD patients and healthy controls, we analyzed microbiome beta diversity using Bray-Curtis distance. Principal coordinate analysis revealed a distinct sample separation along the disease-health axis independent of disease type (Fig. 1b). Using a multivariate statistical approach with medication, age, and sex as covariates, we confirmed the microbiome differences between healthy and disease subjects (PERMANOVA FDR < 0.05, Additional file 1: Table S3). Cohort determination (IgG4-RD, SSc, or healthy control) had the strongest effect on the microbiome variation between the subjects, with 3.6% explained variance (Additional file 1: Table S3). Additional PERMANOVA analysis for IgG4-RD patients only revealed no significant differences in the microbiomes of patients with active disease relative to those of patients in remission (Additional file 1: Table S3). Altogether, our diversity analysis revealed a significantly altered microbiome architecture in IgG4-RD and SSc relative to healthy controls. In the case of SSc, this confirms earlier gut microbiome studies that employed 16S rDNA sequencing [22, 37]. Importantly, the clustering of samples from IgG4-RD and SSc based on their microbiome diversity strongly suggests that they share common microbial signatures.

### Depletion of Bacteroidetes and overabundance of Firmicutes in disease microbiomes

**Fig. 2:**
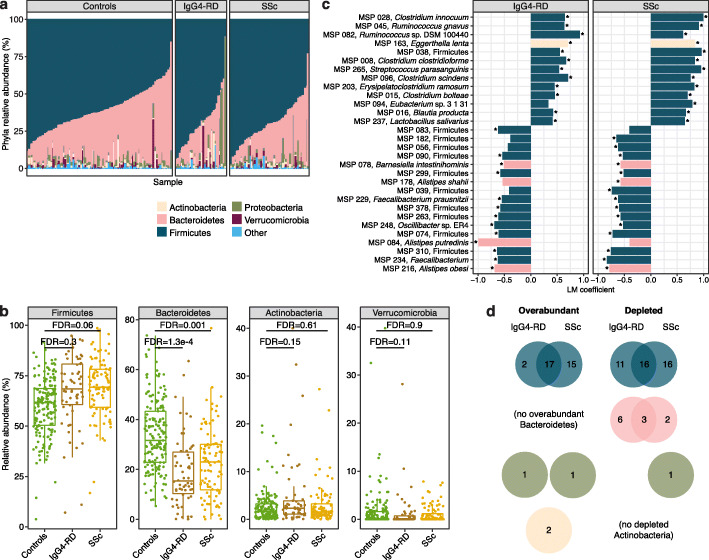
Overabundant and depleted gut microbiome taxa in IgG4-RD and SSc. **a** Relative abundances of the five most abundant phyla in the disease and control cohorts. **b** Differentially abundant phyla in at least one of the disease cohorts when compared to the controls (FDR < 0.2). Boxplots show median and lower/upper quartiles; whiskers show inner fences. **c** Top 30 differentially abundant species in IgG4-RD and/or SSc when compared to the controls (*FDR < 0.05). See also Additional file 1: Table S4. **d** The number of overabundant or depleted species from each phylum in the disease cohorts. In total, 19 overabundant and 19 depleted species were common to both IgG4-RD and SSc; no discordant species (i.e., overabundant in one disease and depleted in the other) were identified. Panels **a**, **c**, and **d** use the same color scheme to represent phyla

To identify specific taxonomic groups that contribute to the reconfigured gut microbiome architecture in these diseases, we tested the association of their relative abundances to disease status using a linear model with age, sex, and medication as covariates. As expected, IgG4-RD, SSc, and control microbiomes were dominated by Bacteroidetes and Firmicutes species (Fig. 2a); however, we saw a consistent depletion of Bacteroidetes in both diseases (FDR ≤ 1 × 10^−3^) with a concordant increase of Firmicutes in SSc (FDR = 0.06) and Actinobacteria in IgG4-RD (FDR = 0.15) relative to controls (Fig. 2b). At the species level, we detected 38 MSPs concordantly overabundant or depleted in both diseases compared to controls (FDR < 0.05, Additional file 1: Table S4, Fig. 2c, d). All 11 differentially abundant Bacteroidetes MSPs were depleted in one or both diseases (Fig. 2d), consistent with our phylum-level observations, and included phylogenetically related taxa (e.g., four *Alistipes* and four *Bacteroides* species). Among the Firmicutes, numerous opportunistic pathogenic *Clostridium* species were overabundant (e.g., *C. innocuum*, *C. clostridioforme*, *C. bolteae*, and *C. symbiosum*). These Clostridia are well-adapted to invade host tissue by encoding pathogenicity factors, such as antibiotic resistance genes and flagellins, and have been identified as extraintestinal infectious agents [38–40]. Additionally, multiple commensals typical of the oral cavity were overabundant in disease, including three *Veillonella* and five *Streptococcus* species. Colonization of the lower parts of the gastrointestinal (GI) tract by oral microbes is a recognized phenomenon in systemic diseases such as RA [41] and has been reported in other immune-mediated diseases such as IBD [42, 43]. In contrast, *Faecalibacterium* species from group IV Clostridium (two MSPs annotated as *Faecalibacterium prausnitzii*) that are known for their beneficial butyrate production were depleted in the two diseases. Lastly, an additional 67 MSPs that were differentially abundant in only one disease showed a consistent trend of overabundance or depletion in the other disease (Additional file 2: Figure S3, Additional file 1: Table S4).

The overabundance of Actinobacteria was attributed to one of the top differentially abundant species, *Eggerthella lenta* (SSc FDR = 5.4 × 10^−7^ and IgG4-RD FDR = 1.8 × 10^−4^). *E. lenta* is also overabundant in the gut microbiome of RA and multiple sclerosis patients [44, 45], suggesting that *E. lenta* might be playing an important role in multiple autoimmune diseases. Apart from the species with known taxonomy, we observed 36 differentially abundant MSPs with no species-level information (FDR < 0.05, Additional file 1: Table S4). Phylogenetic analysis [34] revealed that most of them represented previously uncharacterized Firmicutes (Additional file 2: Figure S4). Among those depleted in disease (33/36 MSPs), we observed that MSP 182, MSP 234, MSP 326, and MSP 378 clustered closely with *F. prausnitzii,* indicating an additional decreased abundance of potential butyrate-producing species. Among those overabundant in either IgG4-RD or SSc, we observed that MSP 038, MSP 122, and MSP 179 formed a closely phylogenetically related clade and as such might encode similar functionalities that allow them to bloom in these diseases or are relevant to the disease phenotype. Finally, we want to highlight that no significant differences in known or unknown MSPs were observed comparing IgG4-RD and SSc patients, reinforcing the view that these diseases share a common microbiome signature.

We next asked if SSc subgroups are characterized by overabundance or depletion of specific MSPs in order to identify potential pathobionts unique to each subgroup. We compared microbiomes in limited cutaneous SSc (lcSSc), diffuse cutaneous SSc (dcSSc), sine scleroderma (ssSSc), and overlap scleroderma (osSSc) to healthy controls. We observed 16 MSPs that were commonly differentially abundant between at least two SSc subgroups; most notably, overabundance of three *Streptococcus* species, *S. parasanguinis*, *S. vestibularis*, and *S. salivarius* was common to lcSSc, dcSSc, and osSSc. Each subgroup also showed unique microbial changes relative to healthy controls (FDR < 0.05, Additional file 1: Table S5, Additional file 2: Figure S5). Depletion of *F. prausnitzii* characterized lcSSc, while overabundance of two taxa typical of the oral cavity, *Veillonella parvula* and *Klebsiella pneumoniae*, was observed in dcSSc. We also tested the pairwise differences between the four SSc subgroups and observed four differentially abundant species in osSSc compared to either lcSSc or dcSSc (FDR < 0.05, Additional file 1: Table S5). Finally, we turned to IgG4-RD patients and tested for species-level differences between patients with active disease or in remission, and observed no significant association.

### Strain-specific gene enrichment in disease

**Fig. 3:**
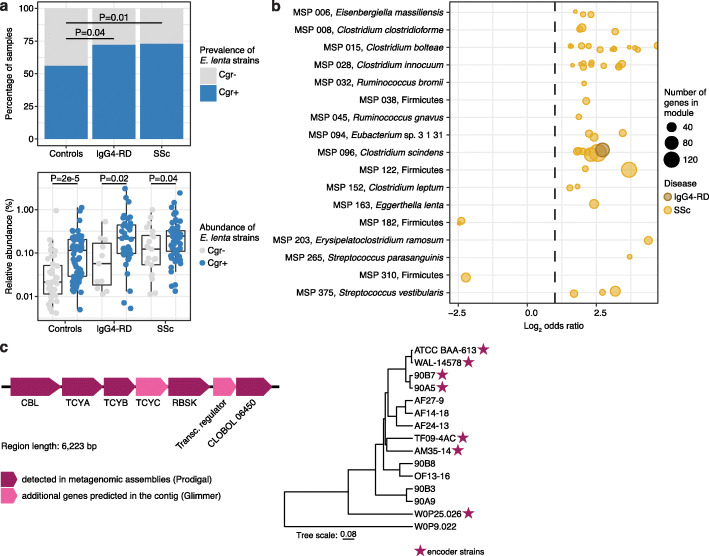
Strain-level signals in IgG4-RD and SSc. **a** Top, Strains of *E. lenta* are more likely to harbor a *cgr* operon (*cgr+*) in disease samples than healthy control samples (Fisher’s test, comparison with controls: IgG4-RD *P* = 0.04; SSc *P* = 0.01; *N* controls = 107, *N* IgG4-RD = 51, *N* SSc = 81). Bottom, *Cgr+ E. lenta* achieve a higher relative abundance than *cgr− E. lenta* in control, IgG4-RD, and SSc samples. Boxplots show median and lower/upper quartiles; whiskers show inner fences. **b** Species-specific accessory modules (acc) that are enriched or depleted in SSc or IgG4-RD compared to healthy controls (FDR < 0.05). Effect size is shown as log_2_ odds ratio. The number of genes contained in each accessory module is indicated by the size of a dot. **c** Accessory module acc 058 in *C. bolteae.* Left, Gene composition of the locus encoding cystine uptake and homocysteine metabolism genes. Right, NCBI’s phylogenetic tree of 15 *C. bolteae* reference genomes. Stars indicate strains encoding the locus

A species-level focus in human microbiome studies can lack the specificity necessary for attributing functional roles, as different strains of the same species can harbor vastly different accessory genomes. These genomic differences can be particularly relevant in the context of immune system activation. For instance, different strains of *K. pneumoniae* show variable potential in activating type 1 T helper (Th) cells, highlighting the importance of accessory genes on modulating immune function [46]. With that in mind, we evaluated the genetic composition of *E. lenta*, which was one of the top differentially abundant species in our comparison (Fig. 2c). *E. lenta* is well-known for its potential to inactivate plant toxins, including the cardiac drug digoxin, in the human gut via a strain-specific cardiac glycoside reductase (*cgr*) operon [47]. The *cgr* locus has been attributed immunomodulatory function: *cgr* locus positive (*cgr+*) *E. lenta* drive activation of Th17 cells and production of pro-inflammatory cytokines, while *cgr*− strains do not show this phenotype [48]. To determine if there is a colonization bias of *cgr+ E. lenta* strains in SSc and IgG4-RD patients, we identified five genes in the assembled gene catalog that comprise the *cgr* locus and evaluated their abundances in each sample. Using that signal as the marker for presence or absence of the *cgr* locus, in samples with sufficient coverage of *E. lenta* (see the “Methods” section), we observed that approximately half of the healthy controls were *cgr+ E. lenta* carriers, consistent with previous reports on the distribution of that function in a general human population [35]. *E. lenta* in patients affected by IgG4-RD and SSc, however, were more likely to be *cgr+* (~ 75% patients; Fisher’s test, IgG4-RD *P* = 0.04, SSc *P* = 0.01, Fig. 3a). Additionally, *E. lenta* tended to reach higher relative abundance in *cgr+* samples compared to *cgr-* samples (Wilcoxon, controls *P* = 2 × 10^−5^, IgG4-RD *P* = 0.02, SSc *P* = 0.04). These observations implicate *E. lenta* as a potential microbiome factor with strain-dependent enzymatic activity that might lead to the breakdown of immune homeostasis and expansion of its population in colonized subjects.

Our assembly-based microbiome characterization through MSPs grouped genes into clusters based on their presence and co-abundance pattern such that each species is represented by its core genes and often multiple clusters of different accessory genes. In order to perform a microbiome-wide screen for disease-related accessory modules, we evaluated whether any specific accessory module was differentially distributed in IgG4-RD and SSc compared to healthy controls. We detected 53 accessory modules (acc) that were enriched or depleted in SSc (FDR < 0.05, Fig. 3b). These accessory modules showed largely similar enrichment or depletion trends in IgG4-RD, however only one was significant in this lower-powered cohort (FDR < 0.05, Fig. 3b). The identified accessory modules contained between 4 and 158 genes that encoded for various, often related functions (Additional file 1: Table S6). The only accessory gene set enriched in both SSc and IgG4-RD, acc 002 from *Clostridium scindens*, contained multiple proteins involved in nutrient transport across membranes (ABC transporters, efflux pumps, and antiporters). In SSc-enriched acc 058 from *C. bolteae*, five of its six genes co-assembled next to each other on the same contig in our samples; two additional genes were predicted in the contig using Glimmer [49]. These similarly co-assemble in a subset of *C. bolteae* reference genomes and represent a variation present only in some strains of this species (Fig. 3c, Additional file 2: Figure S6). Interestingly, this accessory module likely expands access of *C. bolteae* to the sulfur-containing amino acid cysteine as it encodes three cystine transporters (homologs to TCYA, TCYB, and TCYC transport system), which is consistent with the ability of *C. bolteae* to bloom with cysteine as a sole carbon source [50]. An additional gene found in this module, cystathionine beta-lyase (CBL), catalyzes the breakdown of the cysteine-related metabolite cystathionine to homocysteine and pyruvate. Increased homocysteine concentration in circulation could contribute to SSc-related vasculopathy, according to a model in which homocysteine inhibits hydrogen sulfide signaling in blood vessels [51].

### Changes to immune signaling and ECM-related microbial functions

**Fig. 4:**
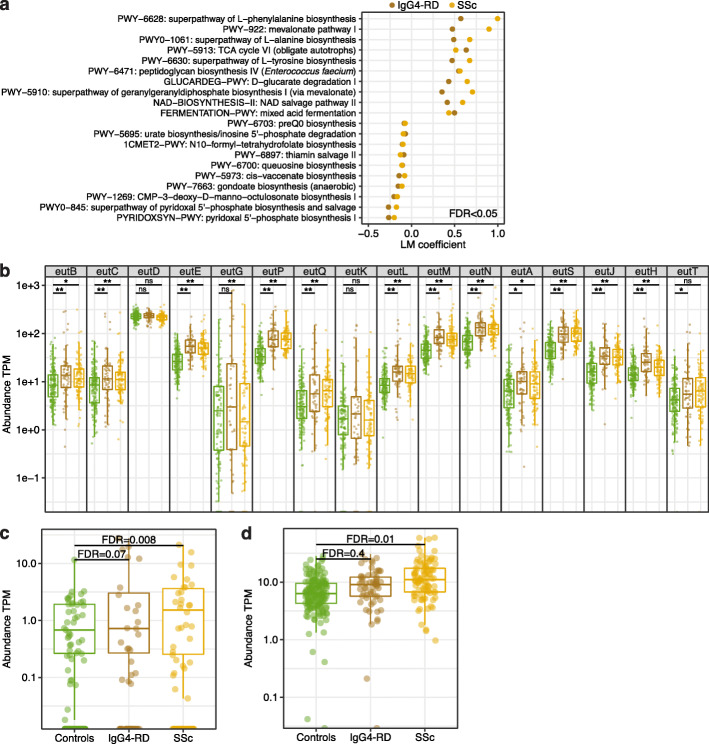
Functional microbiome signatures common to IgG4-RD and SSc. **a** Top 20 significantly up- and downregulated MetaCyc pathways common to IgG4-RD and SSc compared to healthy controls (FDR < 0.05). **b** Numerous genes belonging to the ethanolamine utilization pathway were increased in abundance in disease. Asterisks (*, **) indicate differential abundance at FDR < 0.2 and FDR < 0.05 levels. Differential abundances (transcripts per million, TPM) of **c** Sfb1 (SSc FDR = 0.008, IgG4-RD FDR = 0.07) and **d** HypD (SSc FDR = 0.01) in IgG4-RD and SSc compared to controls. Boxplots show median and lower/upper quartiles; whiskers show inner fences. Full list of differentially abundant MetaCyc pathways and KEGG KO genes are in Additional file 1: Tables S7 and S9

In addition to strain-specific functional potential, multiple species can contribute to similar functional capabilities. To investigate this in an untargeted manner, we summarized the relative abundance of microbial pathways using HUMAnN2 [52]. Consistent with the overall reconfiguration of the microbiome composition in IgG4-RD and SSc, a large number of MetaCyc database pathways were differentially abundant (FDR < 0.05, Fig. 4a, Additional file 1: Table S7). Among the top pathways overabundant in IgG4-RD and SSc were those involved in lipid biosynthesis, including the classical mevalonate pathway (IgG4-RD FDR = 0.003, SSc FDR = 8.6 × 10^−10^) that is encoded by specific microbial families [53]. This overabundance signal was consistent when we investigated the eight enzymes that constitute this pathway (Additional file 1: Table S8). The classical mevalonate pathway leads to the production of isopentenyl pyrophosphate (IPP), a building block for membrane lipids and an important signaling molecule to immune cells [54].

Many biologically important genes are not annotated as pathways; hence, we also evaluated abundance of genes from the assembled gene catalog that were annotated using eggNOG-Mapper [32]. Intriguingly, 12 genes belonging to the ethanolamine utilization compartment were overabundant in IgG4-RD and SSc compared to healthy controls (FDR < 0.05, Fig. 4b). Ethanolamine is particularly prevalent in the GI tract where it is released as a phosphatidylethanolamine from the renewing epithelium, and its abundance increases during inflammation, as observed in IBD [55, 56]. Importantly, ethanolamine can be used as a carbon and nitrogen source to give a growth advantage to the microbes capable of metabolizing it, providing a plausible explanation for the overabundance of potential ethanolamine metabolizers observed in this study, such as *R. gnavus*, *Blautia producta*, or *C. clostridioforme* (Additional file 2: Figure S7).

Finally, we specifically focused on relative abundance changes in functions related to extracellular matrix (ECM) binding and fibrosis. Using eggNOG-Mapper [32], we detected the fibronectin-binding gene *sfb1* (K13734) to be overabundant in SSc and IgG4-RD compared to healthy controls (FDR < 0.1, Fig. 4c). Fibronectins, among others, bind to ECM that is excessively produced during fibrosis, and many pathogens including streptococci use fibronectins as anchors to the epithelium [57, 58]. We also evaluated changes to the recently described 4-hydroxyproline dehydratase (HypD) that degrades the major constituent of ECM, hydroxyproline, and provides access to this nutrient as an energy source for the encoding bacteria [59]. As a relatively novel functional category, it was missing from the annotations obtained using eggNOG-Mapper. We directly searched for homologs of HypD from *Clostridioides difficile* (A0A031WDE4) in the assembled gene catalog and detected 17 homologs with at least 62% amino acid identity [60]. The combined relative abundance signal of the identified HypD homologs was increased in SSc compared to healthy controls (FDR = 0.01, Fig. 4d), which may be reflective of overabundant matrix components in this fibrosis-prone autoimmune disease.

## Discussion

Here we characterized a strikingly similar gut microbiome architecture between two fibrotic autoimmune diseases: IgG4-RD and SSc. Consistent sampling, data generation, and processing allowed us to avoid technical biases and identify biologically relevant features common between the disorders. The gut microbiomes in both diseases were significantly different from healthy controls and showed a depletion of typical, health-associated commensals and expansion of potentially pathogenic and pro-inflammatory species.

Our analysis greatly expanded the number of samples analyzed and added species-level and functional depth to a previous 16S rRNA-based report on the SSc microbiome [37]. Consistent with this study, we observed a depletion of numerous *Bacteroides* and *Faecalibacterium* species as well as an overabundance of *Bifidobacterium dentium* and several *Lactobacillus* species. We additionally identified different *Clostridium* and *Streptococcus* species to be overabundant in SSc compared to healthy controls, a signature that indicates either fewer GI symptoms in our cohort or a cohort-specific signal [14, 37]. In contrast to these previous studies, we directly modeled the contribution of potential confounders, including non-antibiotic medications, when identifying significant associations, which is important for disentangling disease-specific effects [61]. We detected a negative effect on alpha diversity from combinatorial treatment with prednisone and DMARDs, mainly mycophenolate mofetil, methotrexate, or hydroxychloroquine. This observation supports previous reports of the gut microbiome being affected by immunosuppressive medications, such as methotrexate [62] and glucocorticoids [63]. Given a lack of dietary information in this cohort, we could not similarly evaluate the expected effect of diet on the gut microbiome.

By employing a de novo assembly approach to study IgG4-RD and SSc patient microbiomes, we pinpointed changes to the as-yet uncultured constituents of the human gut and performed strain analysis focused on the variation in accessory gene content. This revealed an enrichment in IgG4-RD and SSc of *cgr+ E. lenta* strains, a clade that increasingly is being attributed pro-inflammatory functions [48]. We further observed preferential colonization of SSc patients with a strain of *C. bolteae* encoding a homocysteine metabolism locus. SSc is associated with vasculopathy that may be promoted by elevated levels of homocysteine in circulation [51], which have been reported in SSc and atherosclerosis. Disease-associated microbiomes also showed changes in relative abundances of lipid metabolism and ECM-modifying enzymes. We detected an increase in the relative abundances of genes encoding an ECM binding protein (*sfb1*) and an enzyme that potentially allows for energy extraction from ECM (HypD homologs). These enrichments might enhance the availability of ECM to specific microbial species for attachment and additional nutrient sources [57, 59].

Several functional signatures link the microbiomes of IgG4-RD and SSc patients to inflammation. The classical mevalonate pathway that leads to IPP synthesis was increased in both diseases. IPP and other metabolites from this pathway are recognized by ɣδ T cells as a part of surveillance against microbial infections [64]; differences in the signaling molecule repertoire may alter immune responses in IgG4-RD and SSc patients. Another unifying functional characteristic that we observed was the expansion of pro-inflammatory, Th17 cell-activating *cgr+ E. lenta* [48]. Th17 cell expansion is often related to breakdown of gut microbiome homeostasis and impaired T regulatory cell activity and has been studied experimentally in IBD [65]. Consistent with an increased abundance of *cgr+ E. lenta*, the levels of circulating Th17 cells are elevated in patients with IgG4-RD and SSc [66, 67]. While there is not yet consensus in the field, mouse models of SSc and cellular assays connect the level of IL-17 signaling with the overproduction of collagen and fibrosis [68–70].

Such disease-relevant taxonomical and functional characteristics encourage further study into the identification of specific microbiome-derived molecules that can drive autoimmune pathologies [71], such as microbiome proteins that molecularly mimic disease-associated autoantigens as reported in RA [72]. To date, no specific autoantigens for IgG4-RD or SSc have been identified to facilitate such a discovery, and this remains a challenge for autoimmune diseases in general. Identification of autoantigens and other immune-centered efforts will be needed to link microbiome changes reported here and elsewhere with immunophenotypes in order to further understand the role of the microbiome in IgG4-RD and SSc.

The shared microbiome signature in IgG4-RD and SSc raises a question about the existence of a more universal microbiome architecture in immune-mediated disorders. We observed expansions of *E. lenta* and taxa typical of the oral cavity that are similar to autoimmune diseases including the neuroimmune disorder multiple sclerosis and RA, a disease with multiple joint pathologies [42, 44, 73]. Comparing the differentially abundant species in IgG4-RD and SSc with those from ulcerative colitis and Crohn’s disease, we observed enrichment in four species typically overabundant in IBD (*S. parasanguinis*, *B. producta*, *Lactobacillus gasseri*, and *R. gnavus)* as well as common signals with 11 out of 42 species depleted in IBD [74]. We observed *Escherichia coli* to be overabundant, albeit weakly, in SSs but not IgG4-RD. This pathobiont has been associated with mucosal inflammation and decreased gut anaerobism in IBD [75]. Gut fibrosis in Crohn’s disease leads to thickening of the intestinal wall and strictures; therefore, we searched for the HypD overabundance signal in this disease [24, 56] but detected no significant associations. Future integrative studies will be needed to present a coherent view of gut microbiome signals broadly implicated in immune-mediated disorders.

## Conclusions

Our characterization of the gut microbiome in fibrosis-prone IgG4-RD and SSc revealed taxonomic and functional gut microbiome features that are common in these two diseases, including reduction of health-associated commensals and expansion of potentially pathogenic and pro-inflammatory species. IgG4-RD and SSc patients showed expansion of a Th17-inducing strain of *E. lenta* that encodes a *cgr* locus, indicating a potential microbiome-driven skewing of the immune cell population in the context of these rare autoimmune diseases. We also found that microbiome changes in IgG4-RD and SSc partially recount observations from other autoimmune diseases and that IgG4-RD- and SSc-specific effects likely further shape the landscape of the associated gut microbiomes.

## Supplementary Information

**Additional file 1.** Supplementary Tables.

**Additional file 2.** Supplementary Figures.

## Data Availability

The sequencing dataset generated and analyzed during the current study is available in the NCBI BioProject under PRJNA615162 (https://www.ncbi.nlm.nih.gov/bioproject/ PRJNA615162) [76]. Correspondence and requests for materials should be addressed to R.J.X.

## Abbreviations

IgG4-RD: Immunoglobulin G4-related disease
SSc: Systemic sclerosis
IBD: Inflammatory bowel diseases
RA: Rheumatoid arthritis
MHC: Major histocompatibility complex
CTL: Cytotoxic T cell
HLA: Human leukocyte antigen
lcSSc: Limited cutaneous SSc
dcSSc: Diffuse cutaneous SSc
ssSSc: Sine scleroderma
osSSc: Overlap scleroderma
MSP: Metagenomic species
DMARD: Disease-modifying antirheumatic drug
GI: Gastrointestinal
Th: T helper
cgr: Cardiac glycoside reductase
acc: Accessory modules
CBL: Cystathionine beta-lyase
IPP: Isopentenyl pyrophosphate
ECM: Extracellular matrix
HypD: 4-hydroxyproline dehydratase
