# Supplementary material for: Congruent microbiome signatures in fibrosis-prone autoimmune diseases: IgG4-related disease and systemic sclerosis
Source: Genome Med. 2021 Feb 28;13:35. doi: 10.1186/s13073-021-00853-7 (PMC7919092; doi:10.1186/s13073-021-00853-7)
Supplement: Supplementary file 2 — Additional file 2. Supplementary Figures. [file 13073_2021_853_MOESM2_ESM.pdf]

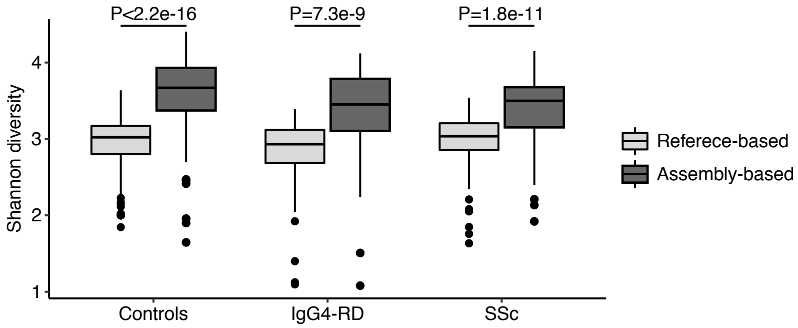

Figure S1. Alpha diversity (Shannon) in cohorts when analyzed using the reference- and assembly-based methods. Comparison of intra-cohort alpha diversities revealed increased alpha diversities using assembly-based methods (Wilcoxon p-values < 10^{-8}). Boxplots show median and lower/upper quartiles; whiskers show inner fences.

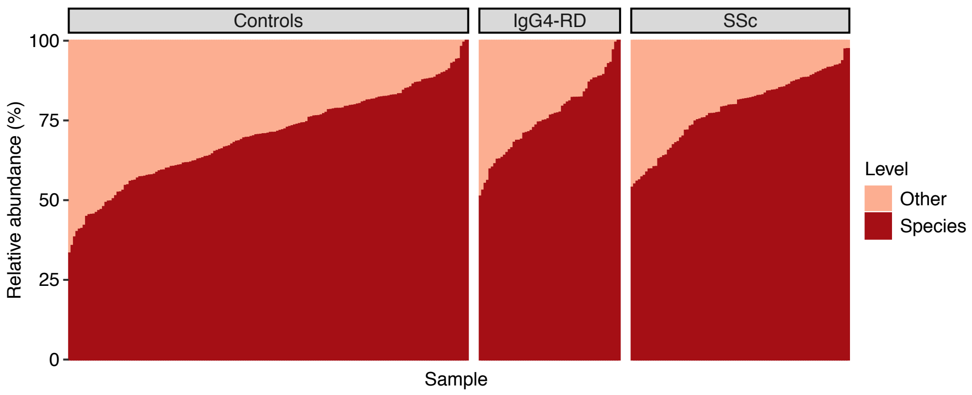

Figure S2. Evaluation of relative abundance contribution for MSPs. In the most extreme cases, over 50% of the signal is contributed by MSPs with no species level resolution.

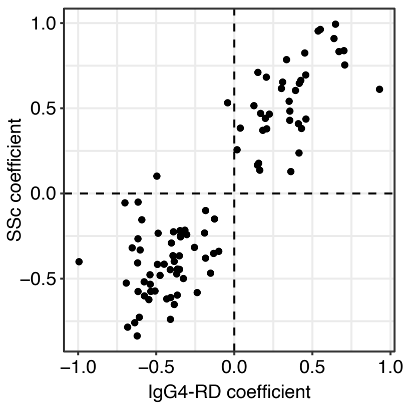

Figure S3. MSPs differentially abundant in IgG4-RD or SSc compared to healthy controls (FDR < 0.05) show coherent up- and down-regulation in both diseases (91/93 MSPs). The two exceptions are MSP 030, *Parabacteroides merdae* and MSP 093, *Lachnospiraceae bacterium* 6 1 37FAA. See also Additional file 1: Table S4.

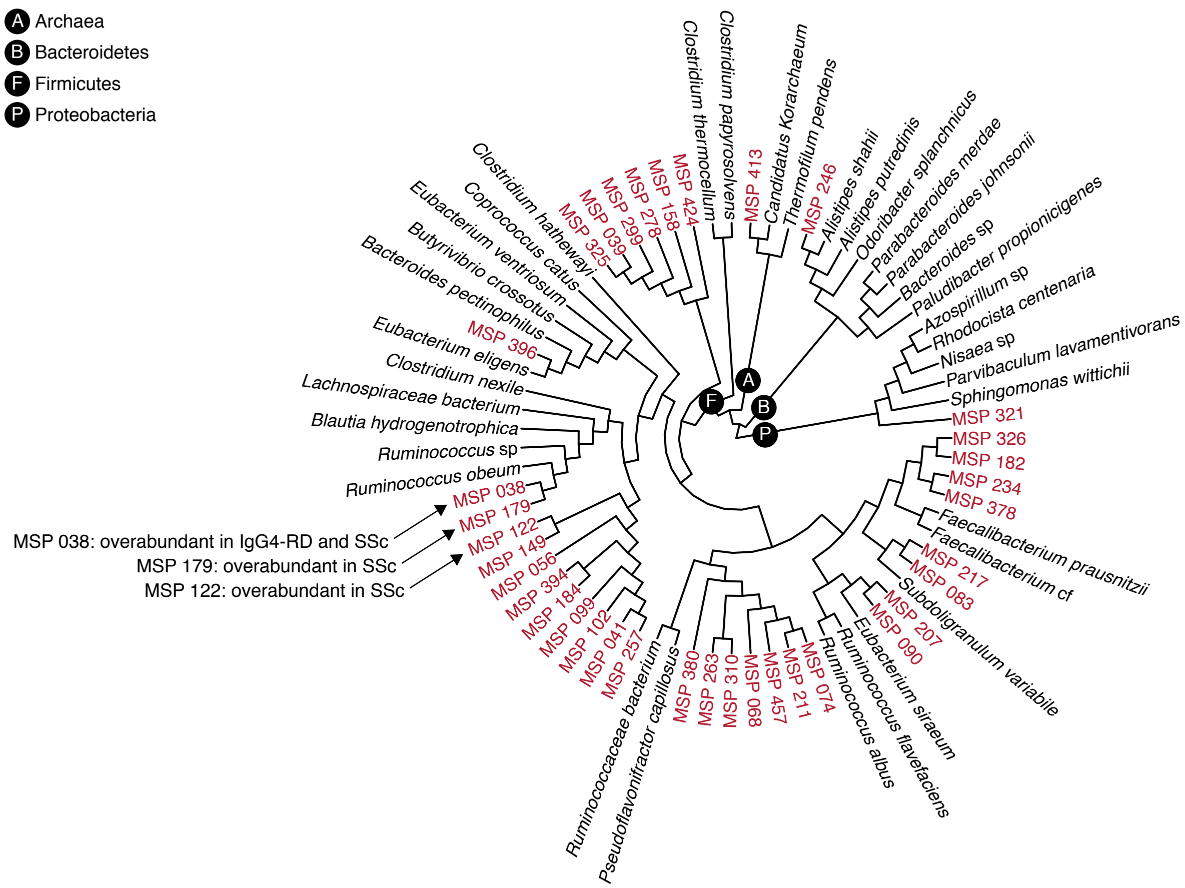

Figure S4. Phylogenetic placement of the 24 MSPs (red) that were significantly differentially abundant between IgG4-RD, SSc, and healthy controls (FDR < 0.05) and that had no species- or genus-level taxonomic annotation. All but the MSPs indicated with an arrow were depleted in IgG4-RD and/or SSc. Legend indicates different bacterial and archaeal phyla; a few known species were plotted in the tree as a reference.

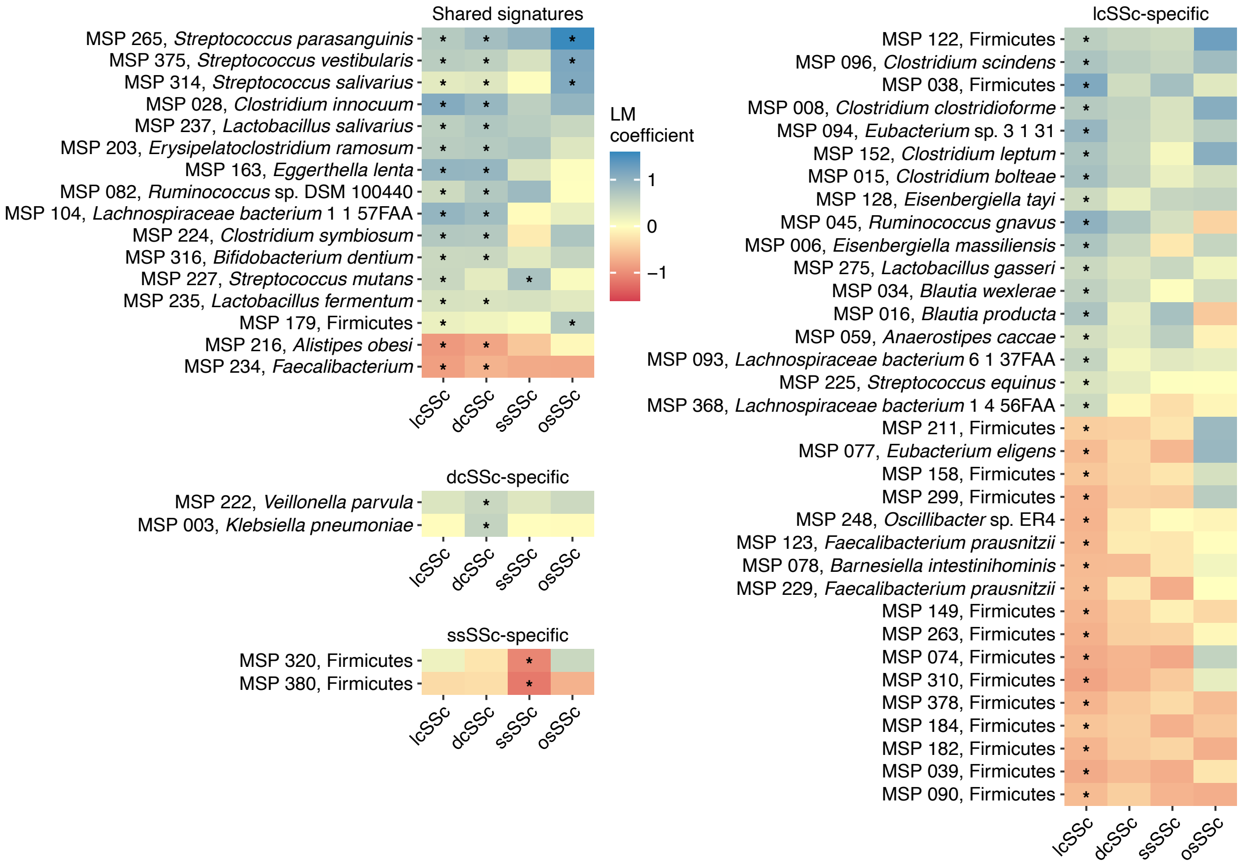

Figure S5. Significant taxa that were differentially abundant in limited cutaneous SSc (lcSSc), diffuse cutaneous SSc (dcSSc), sine scleroderma (ssSSc) and overlap scleroderma (osSSc) relative to healthy controls (FDR < 0.05). See also Additional file 1: Table S5.

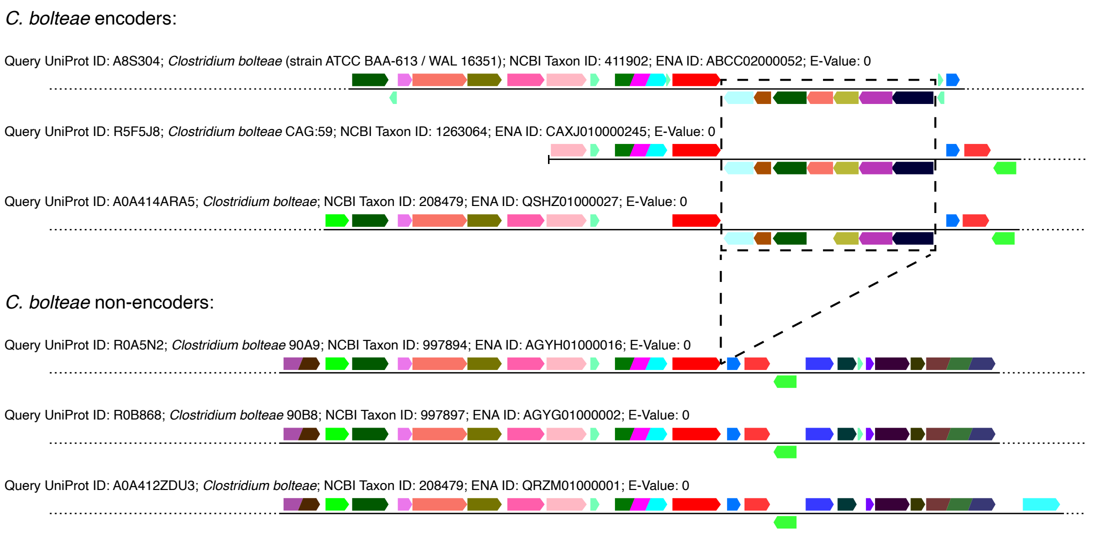

Figure S6. Gene neighborhood in *C. bolteae* encoding and non-encoding strains for the 6,223 base pair locus shown in detail in Figure 3c. Dashed lines indicate the locus and its location. Homologous genes in different genomes are indicated with the same color. Visualization was created using EFI (https://efi.igb.illinois.edu/efi-gnt/).

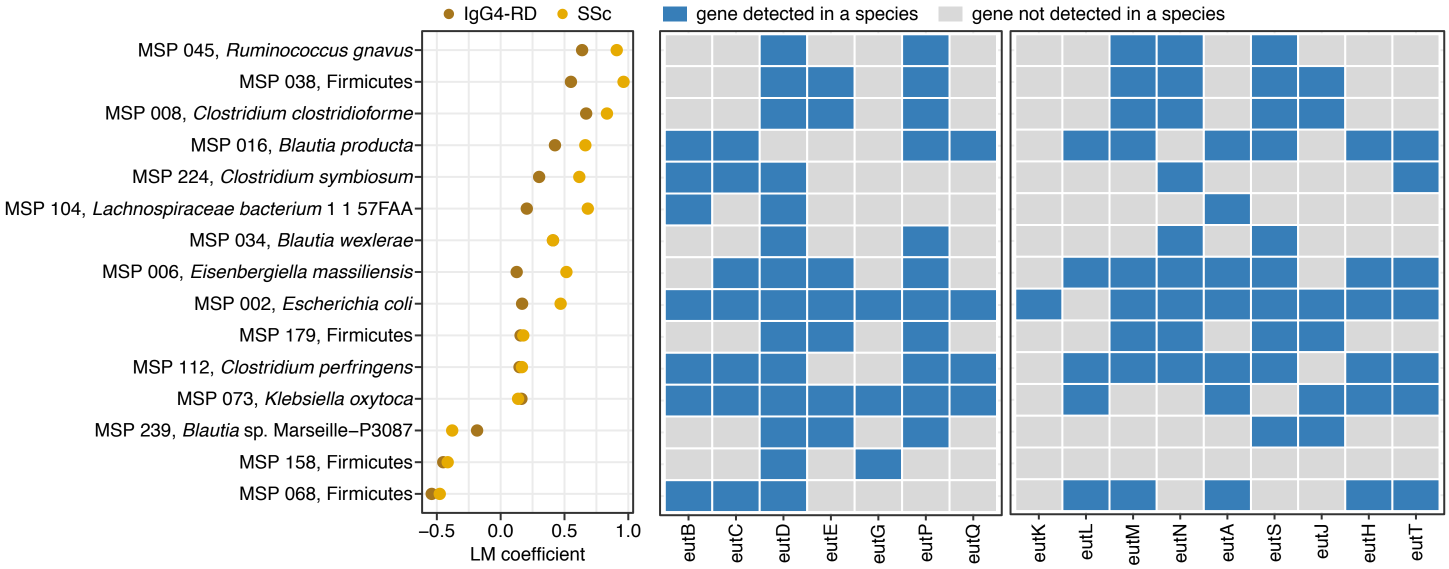

Figure S7. Species encoding at least two genes from the ethanolamine utilization locus. Genes are ordered according to enzymatic steps of ethanolamine utilization (eutB-eutQ) and structural proteins that create a microcompartment for the process to take place in (eutK-eutT).
